# Copper and other heavy metals in grapes: a pilot study tracing influential factors and evaluating potential risks in China

**DOI:** 10.1038/s41598-018-34767-z

**Published:** 2018-11-27

**Authors:** Xiaomin Li, Shujun Dong, Xiaoou Su

**Affiliations:** Institute of Quality Standard and Testing Technology for Agro-Products, The Chinese Academy of Agricultural Sciences (CAAS), Beijing, 100081 China

## Abstract

In this study, grapes (*Vitis vinifera L*.) were systematically sampled across the main grape-producing areas in a nationwide survey of China. Grapes from special regions, such as heavy metal polluted areas (e-waste dismantling area) and pesticide free areas (courtyard) were also collected to make a comparison. Grape skins and pulps were separated to evaluate influence of accumulation behavior, environmental transport and water cleaning efficiency to heavy metals. Levels of copper in grape skins (5.02 ± 3.18 μg/g) were higher than in pulps (3.74 ± 1.48 μg/g). Only high level of copper in two grape skins (sampled from an e-waste dismantling area) showed obvious decrease during water clean-up procedure, indicating the influence of air deposition. Statistical analysis showed no significant difference in the copper levels of grapes from markets, courtyards and e-waste dismantling areas. Concentrations and sources of chromium (Cr), manganese (Mn), nickel (Ni), cadmium (Cd), lead (Pb) and arsenic (As) were also analyzed. Higher levels of these heavy metals were observed in grape skins than pulps. Finally, we evaluated the risk of ingesting heavy metal through grapes using the estimated daily intake (EDI). No health risk was found by consuming grapes according to the data from this study.

## Introduction

Grape (*Vitis vinifera L*.) is one of the most widely consumed fruits around the world. It is not only a popular table fruit, but is also the raw material to produce wine worldwide. Recent studies have suggested that the good cardiac health condition of inhabitants around the Mediterranean area was related to rich content of phenolic compounds in grapes from their diet^1,2^, which might further promote grape consumption. As known, viticulture will be abundantly applied with copper-based pesticide, because this kind of plants is vulnerable to fungal diseases (e.g. downy mildew). In order to control downy mildew, about 400–800 kg per hectare of Bordeaux mixture (mixture of quicklime and copper sulfate, the concentration of copper is about 2.55 g/L) was sprayed several times during the whole grape growing season^3^. During the process of which, the sprayed copper pesticide can be deposited on the fruit surfaces and might find its way into the fruit tissues. Soil in vineyards was also considered to be a seriously copper polluted area due to year by year application of Bordeaux mixture^4–7^. The copper could also be able to transport to grapes through root adsorption. To limit copper concentration, the dose of copper in vineyard soil is limited to 8 kg Cu per hectare in vine cultivation^8^.

Normally copper is nontoxic to mammals^9^, and copper deficiency could even result in inflammation, anemia and other discomforts^10^. However, excessive digestion of copper might cause a series of health problems. Several studies have revealed that too much copper accumulating in human bodies could result in liver cirrhosis, cell hemolysis, anemia, kidney disorders and osteoporosis^11–16^. As such, grapes and grape products that are susceptible to copper might arouse public health concern due to the universal application of copper-based pesticides in viticulture^1,17–20^. Some of these previous studies focused on whether the heavy metal contents exceeded the tolerable daily intake (TDI)^21,22^, and some of them discussed copper distributions in underground and aerial parts of the plants^23^, but few of them discussed the influence of pesticides application or other potential factors (such as environmental pollution) on grapes. In this study, we not only sampled grapes from the main grape-producing areas, but also from e-waste dismantling areas with environmental heavy metal pollution and from home courtyard with pesticide-free situation. These sampling areas allow us to compare and analyze copper and other heavy metals in grapes across Chinese market.

Some of the heavy metals are well known for their toxicity, non-biodegradable nature and potential bioaccumulative behavior in human tissues^13,24,25^. It has been demonstrated that excessive intake of heavy metals could result in damage to organs and cause numerous diseases, which were characterized by osteomalacia, renal and nerve dysfunction, cardiovascular problems and so on^13,26^. The main exposure routes to heavy metals for humans are through dietary. Therefore, the tolerate daily intake of these elements in food were strictly restricted by authorities and countries^27,28^. European Commission (EC) have set up regulations to control lead (Pb) and cadmium (Cd) in foodstuffs^29^, and World Health Organization (WHO) also assessed heavy metal toxicity and recommended ingestion ranges to certain amounts^30^. As an important part of dietary, it is necessary to evaluate, monitor and control heavy metals in fruits.

Copper-based pesticide is intensively used in viticulture, but few studies have evaluated heavy metals in grapes with regard to the large-scale application of Cu-based pesticide. In this study, grapes from markets, home yards and e-waste dismantling areas were collected. The major objectives of this article were to assess the levels of copper in grapes across Chinese market and discuss the influential factors. Moreover, six more important heavy metals (chromium (Cr), manganese (Mn), nickel (Ni), metalloid arsenic (As), Cd and Pb were simultaneously analyzed. Grapes were separated into skins and pulps for analysis respectively. Factors such as pesticide application, soil pollution and water clean-up process were taken into consideration to trace the sources and fates of heavy metals in grapes. Further, the potential health risk of ingesting heavy metals by consuming grapes was also evaluated.

## Results

In this study, contents of heavy metals in samples were expressed on dry weight base. The moistures of grape skins and pulps were in the range of 74.2–88.9% and 78.4–92.3%, and the geometric means were 82.8% and 85.2% respectively. The concentrations of copper in grape skins and pulps were in the range of 1.83–20.1 μg/g dw and 1.27–7.74 μg/g dw, with average mean values of 5.22 μg/g dw and 3.73 μg/g dw, respectively.

Seven grape samples were collected from Taizhou. The mean values of copper in these 7 grape skins and pulps were 5.30 μg/g and 3.10 μg/g respectively, which were in the same level to the average of all samples. Four home grown grapes (pesticide free) showed mean concentrations of copper in skin and pulp to be 5.34 μg/g and 3.21 μg/g respectively, which also were comparable to the mean values obtained from all grape samples.

**Figure 1:**
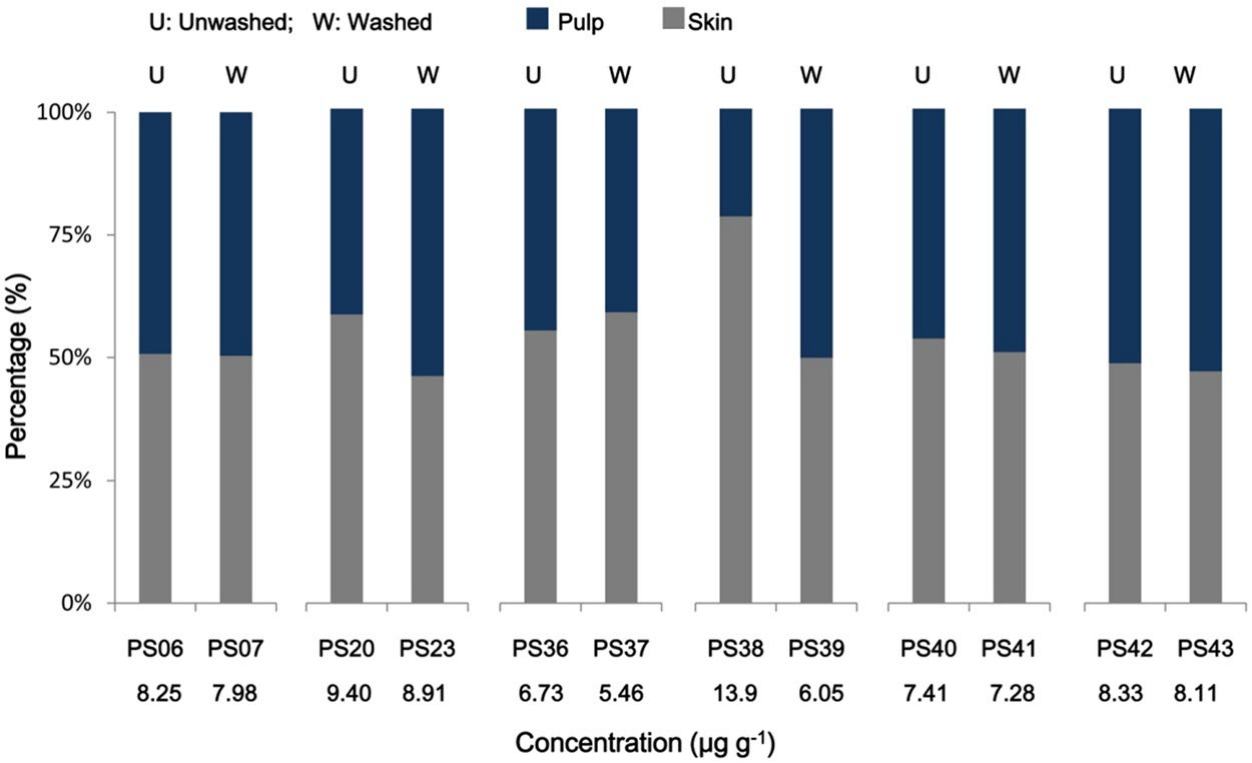
Comparison of copper in paired unwashed and washed grape samples. Data shown below sample name were the total copper concentration in grapes.

The water clean-up process was conducted. The differences of copper levels in paired unwashed and washed grape skins and pulps were shown in Fig. 1, and significant difference has been observed (p < 0.05). Copper levels in all the unwashed skins were higher than in the corresponding washed skins, especially for two paired samples (PS20 and PS23, PS38 and PS39). There were 25.4% and 72.3% of copper removed in PS23 and PS39 respectively, which indicated that a certain amount of copper was attached on the grape skin surface and could be washed away. Both of the two grapes were collected from e-waste dismantling areas.

**Figure 2:**
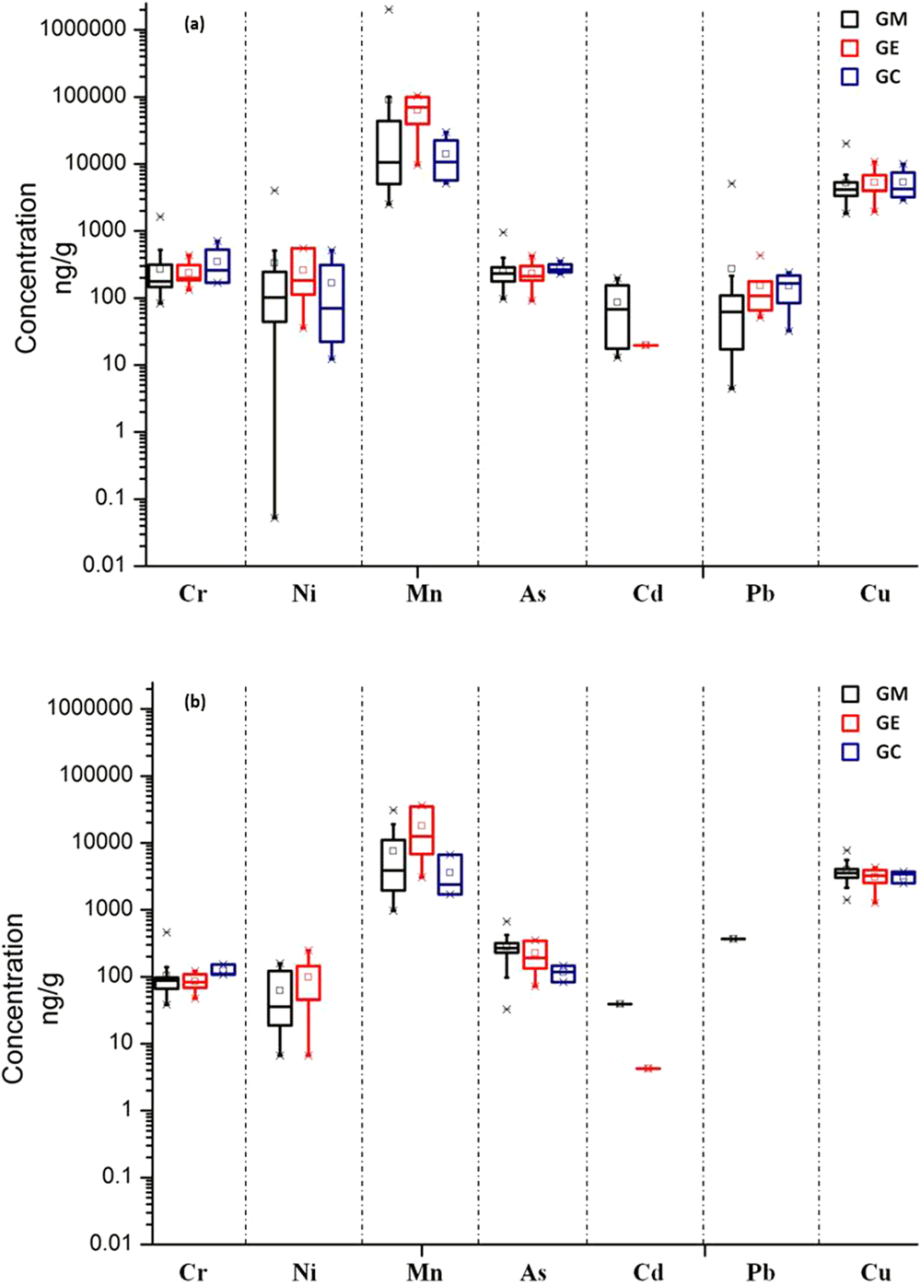
Box-and-whisker plots of Cr, Ni, Mn, As, Cd, Pb and Cu levels in grape (**a**) skins and (**b**) pulps. The box upper and under lines represent 25th and 75th percentiles, and three horizontal bars represent 5th, 50th, and 95th percentiles, *represent the 1% and 99%, represent mean values. GM, GE and GC represent grapes collected from markets, grapes collected from e-waste dismantling areas and grapes collected from courtyards, respectively.

The concentrations of Cr, Mn, Ni, Cd, Pb and As in grape skins (n = 41) and pulps (n = 41) from different functional zones were summarized in Table 1. The average concentrations of all the detected heavy metals in grape skins were higher than in pulps (Fig. 2). Considering the detection frequencies of the rest metals, Cr, Mn and As were 100% detected both in grape skins and pulps. 93.6% of Ni in grape skins and 51.2% of Ni in grape pulps were detected respectively. Geometric means of skin/pulp ratios were 2.15, 3.25, 4.58, 1.01 for Cr, Mn, Ni, As, respectively.

**Table 1:** Levels of heavy metals in grape skins and pulps from different sampling area in China.

Grapes (dry weight)															
		Skins (μg/g)^a^							Pulps (μg/g)						
		Cr	Mn	Ni	Cu	As	Cd	Pb	Cr	Mn	Ni	Cu	As	Cd	Pb
Chinese market (n = 29)	Max	1.64	2013	4.00	11.6	0.95	0.20	5.11	0.46	31.0	0.16	7.72	0.67	0.04	0.37
	Min	0.08	2.51	N.D.^b^	1.83	0.1	N.D.	N.D.	0.04	0.97	N.D.	1.41	0.03	N.D.	N.D.
	Mean^a^	0.26	92.4	0.34	4.67	0.25	0.09	0.27	0.10	7.71	0.06	3.84	0.28	0.04	0.37
E-waste dismantling area (n = 7)	Max	0.44	104	0.56	10.8	0.43	0.02	0.43	0.12	36.1	0.25	4.29	0.35	0.004	N.D.
	Min	0.13	9.71	0.04	1.96	0.09	N.D.	0.05	0.05	3.07	N.D.	1.27	0.07	N.D.	N.D.
	Mean	0.24	63.4	0.26	5.30	0.23	0.02	0.15	0.09	18.0	0.10	3.10	0.23	0.004	N.D.
Courtyard (n = 4)	Max	0.71	29.7	0.52	9.96	0.36	N.D.	0.24	0.15	6.65	N.D.	3.71	0.15	N.D.	N.D.
	Min	0.17	5.12	0.01	2.89	0.23	N.D.	0.03	0.11	1.70	N.D.	2.5	0.08	N.D.	N.D.
	Mean	0.35	14.1	0.17	5.34	0.28	N.D.	0.15	0.12	3.58	N.D.	3.21	0.12	N.D.	N.D.
Chile (n = 1)		0.39	10.4	0.05	20.1	0.21	N.D.	N.D.	0.10	2.65	N.D.	7.74	0.30	N.D.	N.D.
Average value (n = 41)		0.27	77.8	0.30	5.22	0.25	0.07	0.24	0.10	9.22	0.07	3.75	0.26	0.02	0.37

^a^Only the detected values were used to calculate mean values.

^b^N.D. = not detected.

## Discussion

Of all the grape samples analyzed, no sample exceeded the maximum residue limit (MRL) of copper restricted by Chinese national tolerance limit of copper in fruit (10 mg/kg)^31^, nor exceeded the restriction of European Commission (20 μg/g)^32^. The mean value of copper concentration in pulps was higher than some of the previous studies (Table 2). Renan reported the average concentration of grape pulps to be 0.94 μg/g dw in Zhejiang Province, China^3^. In Spain, Olalla *et al*. reported that the copper concentration in grape was 0.52 μg/g ww^1^. However, copper contents in our study were not as high as the grapes obtained from Italy farms (11.32 ± 8.61 μg/g dw)^18^. In order to evaluate the influence of copper-based pesticide on copper levels, we further summarized the copper levels in some other fruits and listed the results in Table S1, Supplementary Information. Some of the listed fruits are sprayed with copper-based pesticide in order to control vine fungal diseases (e.g. apple), and some of them were copper-pesticide free fruits. There were no orders of magnitude difference observed in copper levels among these fruits, expect the fruits from India. And according to these works, concentrations of copper in grapes were in the similar level compared to the other fruits (Table S1, Supplementary Information).

**Table 2:** Concentrations of Copper in grapes around the world.

Country	Grapes	Cu concentrations			Literature
		min	max	mean	
Slovenia	red grapes	1.1 μg/g dw	10.2 μg/g dw	4.2 μg/g dw	^52^
	white grapes	1.6 μg/g dw	8.1 μg/g dw	4.7 μg/g dw	
Spain	red grape	—^a^	—	0.49 μg/g ww	^1^
	white grape	—	—	0.53 μg/g ww	
Egypt	grapes	5.07 μg/g dw	9.15 μg/g dw	7.75 ± 0.90 μg/g dw	^43^
Italy	red grape	—	—	11.3 ± 8.61 μg/g dw	^18^
	white grape	—	—	7.54 ± 7.50 μg/g dw	
France	grapes	—	—	4.5 μg/g dw	^23^
Germany	grape	6.56 μg/g	19.9 μg/g	—	^53^
Turkey	grapes	—	—	5.7 ± 0.1 μg/g dw	^54^
Ukraine	grapes	1.47 μg/g dw	1.72 μg/g dw	—	^55^
China	grape	3.45 μg/g	9.16 μg/g	4.80 μg/g	^56^
India	grapes	—	—	84.4 ± 0.68 μg/g dw	^57^
China	grape skins	1.82 μg/g dw	20.1 μg/g dw	5.02 ± 3.18 μg/g dw	this study
	grape pulps	1.27 μg/g dw	7.74 μg/g dw	3.74 ± 1.48 μg/g dw	this study

^a^Data not available.

**Figure 3:**
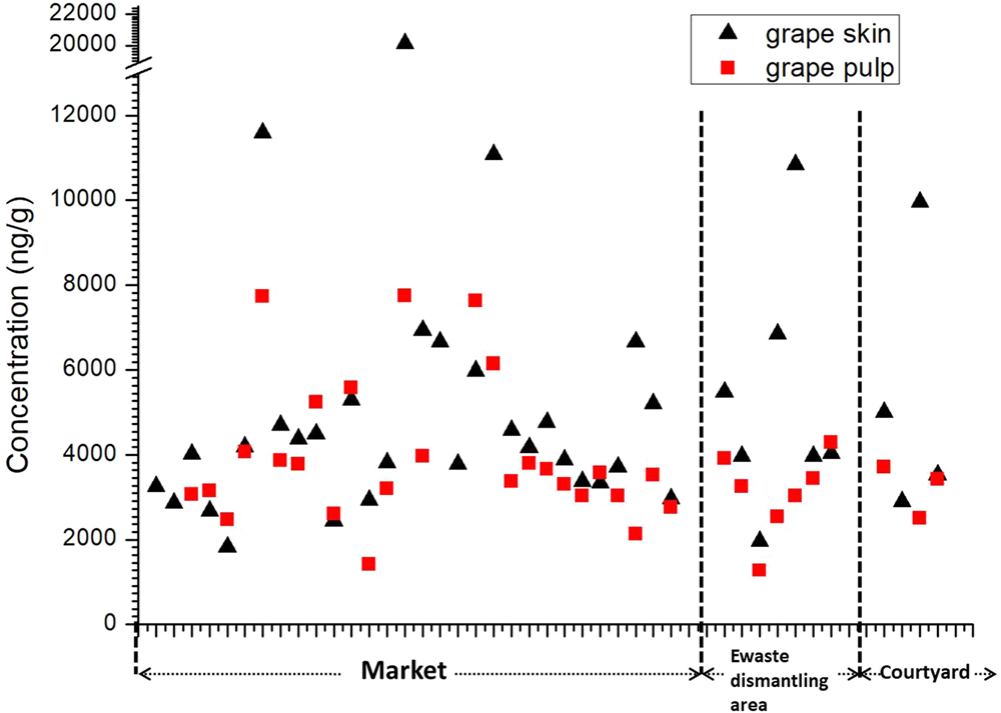
Levels of copper in grape skins and pulps.

Concentrations of copper in grape skins and pulps from markets, ewaste dismantling area and courtyards were shown in Fig. 3. According to the result, copper concentrations in skins were significant higher than in pulps (p < 0.01), and the geometric mean of skin/pulp ratio (n = 41, not including washed samples) was 1.54. The result suggested that pesticide spray and air deposition might result in higher levels of copper in grape skins, or there were distinctive absorption behaviors for grape skins and pulps. A previous work pointed out that pesticide spray was the main source of copper in grapes^18^. However, it still need more evidence to discuss the influences of the other factors beyond application of pesticides.

In this study, we collected grape samples from an intensive e-waste dismantling area to find out whether the environmental contamination (soil uptake or air deposition) would bring heavy metal pollution into grapes or not. Taizhou is a notorious and intensive e-waste dismantling center which located in Southeast China. The e-waste dismantling history of local area can be traced back to 1980s, almost focusing on scrapped transformers and other electrical equipment. Due to the dismantling activities, local soil was seriously polluted by heavy metals. However, not as expected, only two grapes had higher copper levels in grapes from e-waste dismantling area. The average level of copper in all 7 grape samples from Taizhou was in the same level compared to the average level of all grapes, implying that soil from e-waste dismantling area did not bring in general copper pollution to grapes when compared to the commercial vineyard. The copper concentration in local soil was more than 100 μg/g according to previous report^33,34^, which were much higher than Beijing soil (29.0 μg/g)^35^. It was reported that the copper concentration would be around an average of 30 μg/g in the unpolluted soils^36^. However, copper level in Taizhou soil was quite comparable to the copper polluted soils in vineyards^37^. It has been reported that the concentrations of copper in grapes would increase with the age of the vines, but even though only few copper were transported from soils to grapes according to our study. Another study supported our suggestion that the amount of copper which was transferred from soil to plant could only explain a small part of the total copper concentration in plants^4^. Considering the air transport pollutions, levels of copper in total suspended particulate (TSP) in e-waste dismantling area (483 ± 167 ng/m^3^) were several times higher than that in Beijing (110 ± 110 ng/m^3^) and Taiwan (198.6 ± 385.4 ng/m^3^)^38,39^. This TSP-copper was also able to absorb on grape skins. Whether the air deposition will bring in heavy metal pollution to grapes will be discussed later in the clean-up procedure of this study.

Four home grown grapes (pesticide free) were collected to make a comparison to the copper-based pesticide applied grapes. None of these four home grown grapes were sprayed pesticides. Relatively higher copper levels in grape skins were found than pulps. It seems like that grape skins play a better enrichment than grape pulps. Furthermore, the similarity of copper levels in home grown grapes and e-waste dismantling area collected grapes showed that the copper levels were not closely connected to the heavy metal levels of soil. From another point of view, our study suggested that grape might not be a good copper accumulation plant, or the middle environmental levels of copper were rich enough for grapes to absorb and combine to the tissue.

In order to investigate whether copper was attached to the surfaces of grape skin or combined to the tissue, we evaluated the level variation of copper before and after fresh water cleaning. 4.12–72.3% of copper (average 7.62%) was removed after cleaning for all the six paired skin samples. As can be seen from Fig. 1, the copper concentrations in unwashed PS38 and PS20 were higher than the other washed samples (PS39 and PS23, respectively). 25.4% and 72.3% copper in mass was removed. The copper contents in the rest 4 paired skin samples were almost in the same level. Considering the copper levels in PS38 and PS20 were higher than the other unwashed grape skins, the clean-up procedure might be effective to remove copper in high content samples. During the grape maturity, copper might permeate into grapes and combine to grape tissues rather than stay on the surface of skins. Therefore, washing process might be helpful to remove the copper attached to the surface of the grapes, but not to the combined-to-tissue copper in grape skins. As both PS38 and PS20 were collected from e-waste dismantling areas, the higher copper found in local TSP might also have influences as aforementioned. Further research is needed to make a confirmation.

The limited changes of copper concentrations after clean-up process in other four paired grapes suggested that the withdrawal period of pesticide was long enough. Generally, in order to ensure the fruit was safe to eat, the application of Bordeaux mixture will be stopped about fifteen to twenty days before the grape was sold on market. Previous investigation also suggested that safety withdraw time for grape and grape product was more than 20 days. Normally 40 to 50 days were completely enough to limit copper concentrations below 20 μg/g even in dry year^18^. The rain will also be helpful to clean copper off from grape skins. Before the grape harvest, it is the most rainy days in China, which is helpful to remove heavy metals from grapes. As a result, although the application of pesticides was overdose from time to time, the attached copper on grape surfaces was not at a high level in most of grapes. However, it should also be noticed that washing may only reduce the levels of metals in a smattering part. Once metals were introduced and distributed into tissues, they were hardly to be thoroughly removed under traditional handling techniques^40^. Paradelo *et al*. investigated the loss of copper-based pesticides during complex physical washing processes, the result of which showed that mean copper loss was 27.0% for the Bordeaux mixture, and the washing time was not quite related^41^.

**Figure 4:**
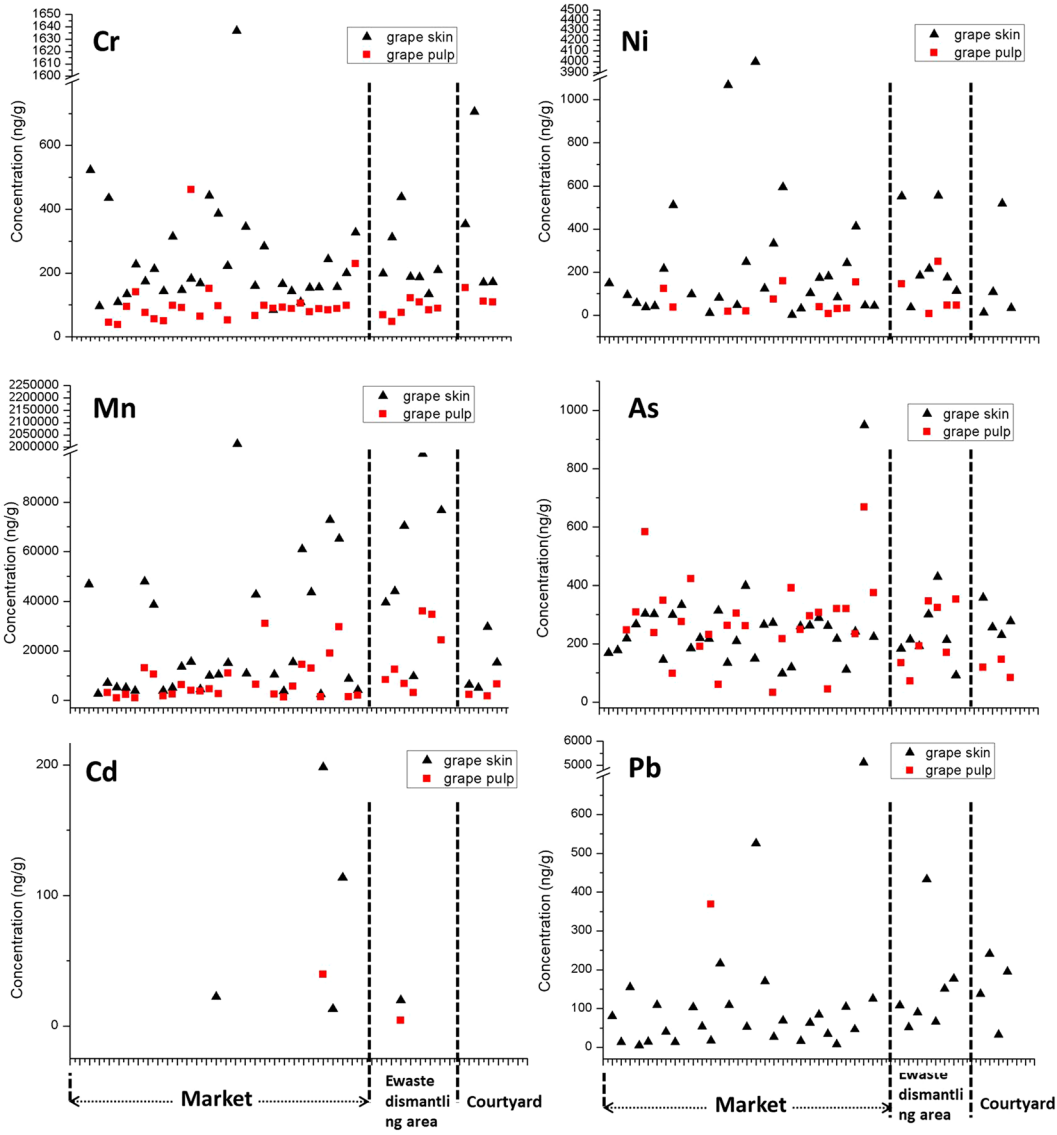
Levels of Cr, Ni, Mn, As, Cd and Pb in grape skins and pulps. The heavy metals under detection limits were left blank.

The other 5 heavy metals and arsenic in grapes were simultaneously analyzed. Figure 4 described concentrations of Cr, Ni, Mn, As, Cd, and Pb in grape skins and pulps from markets, ewaste dismantling area and courtyards, respectively. Level of Mn was found to be the highest among all the detected metals. The average concentrations of Cr, Mn, Ni and Cu in our study were one order of magnitude higher than a national investigation among French markets^42^. Wilcoxon nonparametric test for matched pairs was conducted to analyze the significant difference of metal levels between grape skins and pulps (Table 3). The result also showed that Cr, Mn, Ni and Cu were significant higher in grape skins than in pulps. It is unable to perform statistical analysis to evaluate this difference of Cd and Pb for their low detection rates. Only 2.44% of Pb was detected in grape pulps, but 95.7% of Pb was detected in grape skins. It is not difficult to deduce that there is a significant difference between grape skins and pulps for Pb. Although Cd was rarely detected both in grape skins and pulps, clearly higher level in grape skin was found in Fig. 4. Not as the same as the other heavy metals, As did not show a significant difference between grape skins and pulps, and the result of which were shown in Fig. 4.

**Table 3:** Statistical comparison of heavy metal distribution between grape skins and pulps.

	Cr	Mn	Ni	Cu	As	Cd	Pb
Z	−4.697	−5.232	−3.516	−3.786	−0.141	—^a^	—
p value	p < 0.05	p < 0.05	p < 0.05	p < 0.05	p > 0.05	—	—

^a^Due to most Cd in grape skins and pulps and Pb in grape pulps were under detection limits, so the test values were not given.

The environmental pollution in the e-waste dismantling area was also evaluated for the other heavy metals to see if it has introduced the trace elements into grapes. The levels of Cr, Ni, As, Cd and Pb in grape skins and pulps from e-waste dismantling area were very close to those collected from market and courtyard. Pb was one of the most toxic heavy metals. Comparable Pb levels were found in our study (0.31 μg/g) and another urban waste dumping area, with an average copper level of 0.4 μg/g in grapes^23^. The Pb in grapes obtained from an Egypt market survey showed similar level (0.16 ± 0.02 μg/g dw), but the level of Cd was one order of magnitude lower (0.002 ± 0.001 μg/g dw)^43^. Without direct pesticide spray for these heavy metals, root absorption from water/soil and air deposition might be the two main sources of heavy metals in grapes. Root absorption is closely associated with heavy metal chemical fractions, which were quite different from one heavy metal to another. Pb is less distributed in exchangeable and soluble fraction^44^, which makes it hard to uptake by plant. In the same time, clean-up experiment showed that Pb levels in unwashed skins were 3.0 times higher than washed skins, indicating 66.7% of Pb was removed after the washing process. Differences in Pb content of unwashed and washed grape skins were also evaluated using the paired t-test. Although no significant difference was found (p < 0.05), the value was very close to it (p = 0.06). So it is conducted that a large portion of Pb may come from air deposition.

In order to evaluate the intake of heavy metals, we introduced estimated daily intakes (EDI) to evaluate heavy metal ingestion in this study. EDIs of heavy metals were calculated by using the average levels of grape pulps and skins. The EDI equals to the metal concentrations in fruit multiplying by a man’s daily intake (presumed to be 500 g). In our study, the daily intake of copper through grapes was in the range of 0.11–0.96 mg, and the mean value was 0.28 mg. This result was lower compared to the WHO guideline, but was higher than an investigation of copper ingestion from fruit by Chinese people^45^. Copper was an essential element which was difficult to establish regulatory guideline, because the dose-response curve of copper was found to be a U-shape^15^. This implied that ingestion of too little or too much copper can both cause adverse health consequences^46^. Based on a variety of analytical data of daily diets, the WHO suggested a dose of 0.7 mg copper for women and 0.8 mg for men per day. This value would totally meet daily requirement of humans. The upper limits of TDI for adults were set to be 10 and 12 mg/day for woman and man respectively^30^. No grape samples exceeded the limitation in our study.

The EDIs of toxic metals, such as Pb, Cd and As, were 0.44, 0.06 and 0.33 μg day^−1^ kg^−1^ bw respectively. Even the maximum daily intakes of Pb, Cd and As were under the TDIs (3.6, 1.0 and 50 μg day^−1^ kg^−1^ bw) set by the Food and Agriculture Organization/World Health Organization (FAO/WHO)^27^. Except one pulp sample with a concentration of Pb reaching 0.37 μg/g dw, all the other pulp samples were below the standards restricting the MRLs to 0.1 μg/g (Pb) and 0.05 μg/g (Cd) in China standard^28^ and European regulations^29^. Excessive ingestion of As is associated with skin and internal cancers, as well as various noncarcinogenic effects^47^. Therefore, the As exposure, which was generally through human diet, caused great public attention and need continuous monitoring. The digestion of As through grapes in the present study were two orders of magnitude lower than the levels of FAO/WHO limit. The results implied that the consumptions of these grapes did not pose a health risk to the consumers, but the contributions from total daily dietary consumption should not be ignored.

## Methods

**Figure 5:**
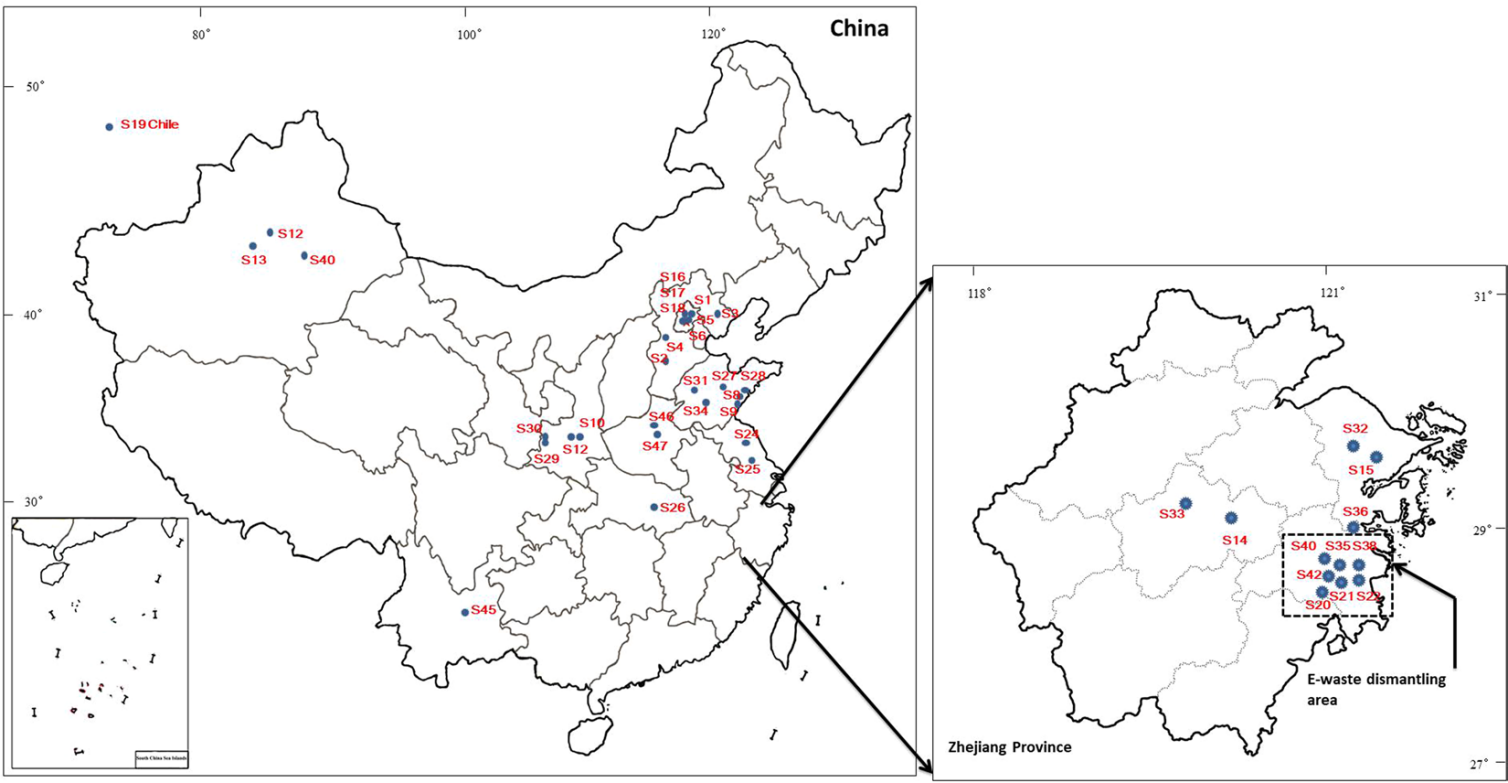
The sampling sites of grapes in China.

Forty grape samples (S1–S41, except for S19) were collected from 11 provinces in China (Fig. 5) during 2009 and 2010. Seven samples were collected from e-waste dismantling areas (S20, S21, S22, S35, S38, S40 and S42) and twenty-nine samples were purchased from local markets (all the grapes were locally grown). The rest 4 samples (S16, S17, S18 and S47) were home grown grapes and collected directly from home courtyard. Moreover, one sample was from Chile (S19). In order to evaluate the influences of copper contents in grapes by water-cleanup process, 6 samples were divided into two equal parts and the 6 half portions were carefully washed by fresh water. S7, S23, S37, S39, S41 and S43 were the washed aliquot parts to S6, S20, S36, S38, S40 and S42. The batch of grape samples are collected from the major grape production areas in China (such as Xinjiang, Shandong and Hebei Provinces). Although the production volumes of grapes increased from 855 tonnes in 2010 to 1120 tonnes in 2017 (nearly 31.0%)^48^, the major grape producing areas did not change a lot over the past decades^49^. Bordeaux mixture is currently employed as a major pesticide for grapes in China for its good efficacy and cost-effectiveness^50^. Heavy metals are known unable to degrade as fundamental elements. Therefore, it indicated that the grape samples collected in the present study were still meaningful nowadays.

The samples were carefully stored and kept in a cooling box packed with ice. The box and its contents were then transported to the laboratory quickly. When the samples arrived, they were manually separated into skins and pulps immediately and stored in a refrigerator under −20 °C for 24 hours. The samples were later lyophilized at −56 °C under a pressure of 0.05 bar. The dry samples were then grinded to fine powders and sealed in polyethylene bags. Both the wet and dry weights of skins and pulps were recorded and the moisture contents were calculated for skin and pulp samples. The moisture content was defined by the following equation^51^:

1 $$MC=\frac{WW-DW}{WW}\times 100 \% $$

where *MC* represents the moisture content, *WW* and *DW* were the wet weight and dry weight of samples.

Approximately 0.2 g dry samples were weighed and carefully transferred to a polytetrafluoroethylene digestion container. Nitric acid (3 mL, Merck, Germany) followed by 30% hydrogen peroxide (3 mL, Beijing Chemical Company, China) were added into the polytetrafluoroethylene container and the mixture was predigested for 3 hours at 60 °C. The mixture was then further digested using a microwave (CEM Mars-X500, USA), with the following program: ramped at 160 °C in 10 min and held for 30 min, then raised to 180 °C in 10 min and held for 30 min. The resultant solutions were then diluted with Milli-Q water to approximate 40 g. The dilution factor could be calculated by the sample weight and the final dilution weight.

The standard solutions were prepared from 100 mg/L stock (Agilent, USA) in 2% nitric acid. The samples were then analyzed by an inductively coupled plasma mass spectrometry (ICP-MS, Agilent 7500, USA). Determinations were performed in triplicate by following previously established methods^45^.

Internal calibration method was used to qualify and quantify real samples. The ranges of our linear standard curves were between 0.5 and 100 ng/mL for all the metals. 94 samples were determined in three batches. In each batch, two blank samples, two standard reference materials (SRM) and 31–32 samples were analyzed. All the 6 blank samples in 3 batches were under detection limits. Two SRMs including peach leaves (GBW08501) and tea leaves (GBW08513) were analyzed to guarantee the analytical procedures. The results showed a good agreement to the certificate values. Except Pb was 70.2 ± 5.1%, the recoveries of Cu, Mn, Cr, Ni and As were in the range of 79.0–83.4%, 96.8–102%, 81.2–89.4%, 79.9–85.7% and 97.0–119%. During the ICP-MS analysis procedure, the analytical accuracy was also evaluated by analyzing 10 ppb standard solution for every 20 samples. The mean value and relative standard deviation (RSD) of copper were 9.93 ng/g and 2.06%, and the RSD of other heavy metals were in the range of 0.9–6.32%.

Statistical analysis was conducted to analyze the distribution and correlation of the studied parameters. The concentration relationships among the detectable heavy metals in grape skins and pulps were analyzed using the nonparametric Wilcoxon test. Paired t-test was conducted to see if there was significant difference between unwashed and washed skins. SPSS 17.0 for Windows (SPSS Inc., 2009) was performed and all the significance level for all the tests was set to p < 0.05 unless otherwise mentioned.

## Electronic supplementary material

Concentrations of copper in fruits around the world.

## Data Availability

All data included in this study are available upon request by contact with the corresponding author.
