# Supplementary material for: Copper and other heavy metals in grapes: a pilot study tracing influential factors and evaluating potential risks in China
Source: Sci Rep. 2018 Nov 27;8:17407. doi: 10.1038/s41598-018-34767-z (PMC6258723; doi:10.1038/s41598-018-34767-z)
Supplement: Supplementary file 1 — Concentrations of copper in fruits around the world. [file 41598_2018_34767_MOESM1_ESM.pdf]

Subject areas: Heavy metals, Grape, Copper-based pesticide application, E-waste dismantling areas

Correspondence and requests for materials should be addressed to Xiaomin Li (lixiaomin@caas.cn)

Copper and other heavy metals in grapes: a pilot study tracing influential factors and evaluating potential risks in China

Xiaomin Li, Shujun Dong & Xiaoou Su*

Institute of Quality Standard and Testing Technology for Agro-Products, The Chinese Academy of Agricultural Sciences (CAAS), Beijing 100081, China

Table S1 Concentrations of Copper in fruits around the world.

Country	Fruit	Cu concentration			Literature
		min	max	mean	
Pakistan	apple			0.50 µg/g dw.	[1]
	mango			1.18 µg/g dw.	
	chilies			1.67 µg/g dw.	
	mint			0.29 µg/g dw.	
Slovenia	red grapes	1.1 µg/g dw.	10.2 µg/g dw.	4.2 µg/g dw.	[2]
	white grapes	1.6 µg/g dw.	8.1 µg/g dw.	4.7 µg/g dw.	
Turkey	fruit	2 µg/g dw.	20 µg/g dw.		[3]
Spain	strawberries			0.58 µg/g ww.	[4]
	oranges			0.87 µg/g ww.	
Spain	red grape			0.49 µg/g ww.	[5]
	white grape			0.53 µg/g ww.	
Poland	pears	0.30 µg/g dw.	2.31 µg/g dw.		[6]
	apples	0.10 µg/g dw.	1.92 µg/g dw.		
	strawberrries	0.229 µg/g dw.	4.74 µg/g dw.		
	black currants	0.097 µg/g dw.	7.07 µg/g dw.		
Egypt	grapes	5.07 µg/g dw.	9.15 µg/g dw.	7.75±0.90 µg/g dw.	[7]
	apples	1.25 µg/g dw.	1.78 µg/g dw.	1.47±0.20 µg/g dw.	
	peach	1.10 µg/g dw.	1.89 µg/g dw.	1.46±0.38 µg/g dw.	
	strawberries	1.32 µg/g dw.	5.79 µg/g dw.	2.17±0.83 µg/g dw.	

Italy	red grape			11.3±8.61 μg/g dw.	8
	white grape			7.54±7.50 μg/g dw.	
Turkey	black olives	0.73 μg/g ww.	2.55 μg/g ww.		9
	green olives	0.54 μg/g ww.	1.37 μg/g ww.		
France	grapes			4.5 μg/g dw.	10
Korea	apples	0.58 μg/g dw.	6.01 μg/g dw.	1.24 μg/g dw.	11
France	fruits			0.65μg/g ww.	12
Germany	grape	6.56 μg/g	19.9 μg/g		13
Turkey	grapes			5.7±0.1 μg/g dw.	14
	apple			5.2±0.1 μg/g dw.	
Ukraine	grapes	1.47 μg/g dw.	1.72 μg/g dw.		15
Algeria	melons			4.00 μg/g dw.	16
	strawberries			4.00 μg/g dw.	
China	jujube	4.1±0.1μg/g dw.	73.6±3.2μg/g dw.		17
China	orange	3.09 μg/g	6.13 μg/g	4.30 μg/g	18
	grape	3.45 μg/g	9.16 μg/g	4.80 μg/g	
	peach-shaped plum	2.99 μg/g	5.62 μg/g	3.92 μg/g	
	pear	3.08 μg/g	7.42 μg/g	4.56 μg/g	
India	banana			33.2±0.12 μg/g dw.	19
	sweet lime			82.2±0.06 μg/g dw.	
	pomegranate			69±0.01 μg/g dw.	
	lychees			36.6±0.09 μg/g dw.	
	mango			85±1.5 μg/g dw.	

	cantaloupe	87±1.8 μg/g dw.	
	watermelon	74±1.1 μg/g dw.	
	papaya	24±0.09 μg/g dw.	
	Indian apple	39.8±0.002 μg/g dw.	
	Australian apple	78.8±0.04 μg/g dw.	
	New Zealand apple	75.4±0.16 μg/g dw.	
	Asian pear (Chinese)	5.06±0.60 μg/g dw.	
	orange	58±0.51 μg/g dw.	
	apricot fresh	72.4±1.3 μg/g dw.	
	plum	81.6±1.0 μg/g dw.	
	sapota	79.4±0.04 μg/g dw.	
	peach (hill)	79.6±0.09 μg/g dw.	
	peach (plane)	66±0.11 μg/g dw.	
	cherry	87.6±0.16 μg/g dw.	
	grapes	84.4±0.68 μg/g dw.	
	green almond	60.2±0.17 μg/g dw.	
	tiger nut	29.4±2.8 μg/g dw.	
	pineapple	54.8±0.64 μg/g dw.	
	guava	62.2±0.96 μg/g dw.	
Bangladesh	banana	0.946±0.043 μg/g dw.	[20]
	jackfruit	11.78±0.419 μg/g dw.	
	mango	7.891±0.307 μg/g dw.	
Cyprus	fig	4.52±0.78 μg/g dw.	[21]

	lemon			4.10±2.45 μg/g dw.	
China	grape skins	1.82 μg/g dw.	20.1 μg/g dw.	5.02±3.18 μg/g dw.	this study
	grape pulps	1.27μg/g dw.	7.74 μg/g dw.	3.74±1.48 μg/g dw.	this study

References

1. Parveen, Z., Khuhro, M. I. & Rafiq, N. Market basket survey for lead, cadmium, copper, chromium, nickel, and zinc in fruits and vegetables. Bulletin of Environmental Contamination and Toxicology 71, 1260-1264(2003).

2. Kristl, J., Veber, M. & Slekovec, M. The contents of Cu, Mn, Zn, Cd, Cr and Pb at different stages of the winemaking process. Acta Chimica Slovenica 50, 123-136(2003).

3. Turkdogan, M. K., Kilicel, F., Kara, K., Tuncer, I. & Uygan, I. Heavy metals in soil, vegetables and fruits in the endemic upper gastrointestinal cancer region of Turkey. Environmental Toxicology and Pharmacology 13, 175-179(2003).

4. Bordajandi, L. R., Gomez, G., Abad, E., Rivera, J., Fernandez-Baston, M. D., Blasco, J. & Gonzalez, M. J. Survey of persistent organochlorine contaminants (PCBs, PCDD/Fs, and PAHs), heavy metals (Cu, Cd, Zn, Pb, and Hg), and arsenic in food samples from Huelva (Spain): Levels and health implications. Journal of Agricultural and Food Chemistry 52, 992-1001(2004).

5. Olalla, M., Fernandez, J., Cabrera, C., Navarro, M., Gimenez, R. & Lopez, M. C. Nutritional study of copper and zinc in grapes and commercial grape juices from Spain. Journal of Agricultural and Food Chemistry 52, 2715-2720(2004).

6. Krejpcio, Z., Sionkowski, S. & Bartela, J. Safety of fresh fruits and juices available on the polish market as determined by heavy metal residues. Polish Journal of Environmental Studies 14, 877-881(2005).

7. Radwan, M. A. & Salama, A. K. Market basket survey for some heavy metals in Egyptian fruits and vegetables. Food and Chemical Toxicology 44, 1273-1278(2006).

8. Garcia-Esparza, M. A., Capri, E., Pirzadeh, P. & Trevisan, M. Copper content of grape and wine from Italian farms. Food Additives and Contaminants 23, 274-280(2006).

9. Sahan, Y., Basoglu, F. & Gucer, S. ICP-MS analysis of a series of metals (Namely: Mg, Cr, Co, Ni, Fe, Cu, Zn, Sn, Cd and Pb) in black and green olive samples from Bursa, Turkey. Food Chemistry 105, 395-399(2007).

10. Chopin, E. I. B., Marin, B., Mkoungafoko, R., Rigaux, A., Hopgood, M. J., Delannoy, E., Cances, B. & Laurain, M. Factors affecting distribution and mobility of trace elements (Cu, Pb, Zn) in a perennial grapevine (Vitis vinifera L.) in the Champagne region of France. Environmental Pollution 156, 1092-1098(2008).

11. Park, B. J. & Cho, J. Y. Assessment of Copper and Zinc in Soils and Fruit with the Age of an Apple Orchard. Journal of the Korean Society for Applied Biological Chemistry 54, 910-914(2011).

12. Noel, L., Chekri, R., Millour, S., Vastel, C., Kadar, A., Sirot, V., Leblanc, J. C. & Guerin, T. Li, Cr, Mn, Co, Ni, Cu, Zn, Se and Mo levels in foodstuffs from the Second French TDS. Food Chemistry 132, 1502-1513(2012).

13. Zhu, F. M., Du, B., Li, F. Y., Zhang, J. C. & Li, J. Measurement and analysis of mineral and heavy metal components in grape cultivars by inductively coupled plasma-optical emission spectrometer (ICP-OES). Journal Fur Verbraucherschutz Und Lebensmittelsicherheit-Journal of Consumer Protection and Food Safety 7, 137-140(2012).

14. Soylak, M., Cihan, Z. & Yilmaz, E. Heavy metal contents of organically produced, harvested, and dried fruit samples from Kayseri, Turkey. Environmental Monitoring and Assessment 185, 2577-2583(2013).

15. Vystavna, Y., Rushenko, L., Diadin, D., Klymenko, O. & Klymenko, M. Trace metals in wine and vineyard environment in southern Ukraine. Food Chemistry 146, 339-344(2014).

16. Cherfi, A., Abdoun, S. & Gaci, O. Food survey: Levels and potential health risks of chromium, lead, zinc and copper content in fruits and vegetables consumed in Algeria. Food and Chemical Toxicology 70, 48-53(2014).

17. Yan, Q. G., Liu, Y. T. & Yang, L. Analysis of Essential and Toxic Elements in Jujube Fruits Collected from Different Locations in China. Tropical Journal of Pharmaceutical Research 13, 607-611(2014).

18. Fang, B. & Zhu, X. Q. High content of five heavy metals in four fruits: Evidence from a case study of Pujiang County, Zhejiang Province, China. Food Control 39, 62-67(2014).

19. Verma, Y. & Rana, S. V. S. Assessment of cadmium, chromium, and copper levels in market fruit samples in Meerut, North India. Toxicological and Environmental Chemistry 96, 1516-1522(2014).

20. Shaheen, N., Irfan, N. M., Khan, I. N., Islam, S., Islam, M. S. & Ahmed, M. K. Presence of heavy metals in fruits and vegetables: Health risk implications in Bangladesh. Chemosphere 152, 431-438(2016).

21. Christou, A., Theologides, C. P., Costa, C., Kalavrouziotis, I. K. & Varnavas, S. P. Assessment of toxic heavy metals concentrations in soils and wild and cultivated plant species in Limni abandoned copper mining site, Cyprus. Journal of Geochemical Exploration 178, 16-22(2017).
